# Acupoint nanocomposite hydrogel for simulation of acupuncture and targeted delivery of triptolide against rheumatoid arthritis

**DOI:** 10.1186/s12951-021-01157-z

**Published:** 2021-12-07

**Authors:** Shujing Ren, Heng Liu, Xitong Wang, Jiquan Bi, Shengfeng Lu, Chenqi Zhu, Huizhu Li, Wenliang Kong, Rui Chen, Zhipeng Chen

**Affiliations:** 1 School of Pharmacy, Nanjing University of Chinese Medicine, Nanjing, 210023 China; 2 Key Laboratory of Acupuncture and Medicine Research of Ministry of Education, Nanjing, 210023 China; 3 Nantong First People’s Hospital, Nantong, 226001 China; 4 Yunnan Provincial Key Laboratory of Entomological Biopharmaceutical R&D, Dali University, Dali, 671000 China; 5 Gusu School, Nanjing Medical University, Suzhou, 215002 China; 6 Jiangyin Hospital Affiliated to Nanjing University of Chinese Medicine, Jiangyin, 214400 China

**Keywords:** Rheumatoid arthritis, Controlled release, Triptolide, Acupuncture

## Abstract

**Background:**

Attenuating inflammatory response and relieving pain are two therapeutic therapeutical goals for rheumatoid arthritis (RA). Anti-inflammatory and analgesic drugs are often associated with many adverse effects due to nonspecific distribution. New drug delivery systems with practical targeting ability and other complementary strategies urgently need to be explored. To achieve this goal, an acupoint drug delivery system that can target deliver anti-inflammatory drugs and simulate acupuncture in relieving pain was constructed, which can co-deliver triptolide (TP) and 2-chloro-N (6)-cyclopentyl adenosine (CCPA).

**Results:**

We have successfully demonstrated that acupoint nanocomposite hydrogel composed of TP-Human serum album nanoparticles (TP@HSA NPs) and CCPA could effectively treat RA. The result shows that CCPA-Gel can enhance analgesic effects specifically at the acupoint, while the mechanical and thermal pain threshold was 4.9 and 1.6 times compared with non-acupoint, respectively, and the nanocomposite gel further enhanced. Otherwise, the combination of acupoint and nanocomposite hydrogel exerted synergetic improvement of inflammation, bone erosion, and reduction of systemic toxicity. Furthermore, it could regulate inflammatory factors and restore the balance of Th17/Treg cells, which provided a novel and effective treatment strategy for RA. Interestingly, acupoint administration could improve the accumulation of the designed nanomedicine in arthritic paws (13.5% higher than those in non-acupoint at 48 h), which may explain the better therapeutic efficiency and low toxicity.

**Conclusion:**

This novel therapeutic approach-acupoint nanocomposite hydrogel, builds a bridge between acupuncture and drugs which sheds light on the combination of traditional and modern medicine.

**Graphical Abstract:**

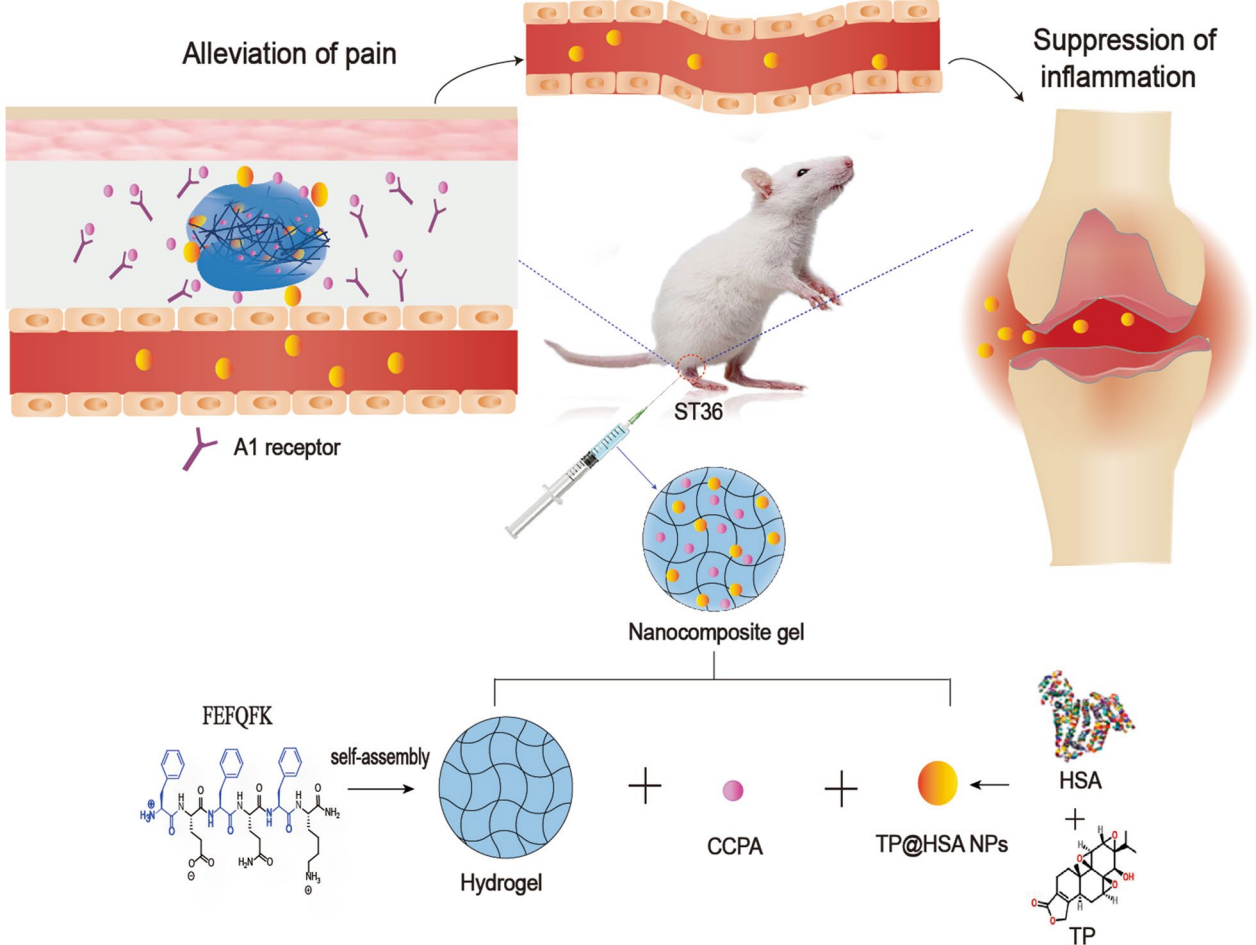

**Supplementary Information:**

The online version contains supplementary material available at 10.1186/s12951-021-01157-z.

## Background

Rheumatoid arthritis (RA) is a common systemic inflammatory autoimmune disease characterized by painful, swollen joints that severely impair physical function and quality of life [1]. Current treatment primarily focuses on the suppression of inflammation and alleviation of pain [2]. Drugs for treating RA mainly include nonsteroidal anti-inflammatory drugs (NSAIDs), disease-modifying antirheumatic drugs (DMARDs), glucocorticoids, and biological agents [3, 4]. The lack of organ/tissue specificity after oral or systemic administration often necessitates higher dosing levels and/or frequency to achieve effective concentrations at sites of inflammation and pathology, further aggravating the adverse side effects [5]. Thus, a rational design of drug delivery systems (DDSs) able to deliver drugs in a controlled manner and for a necessary period to the arthritis joints is a key in developing safe and effective formulations for RA.

Numerous drug delivery systems like nanoparticles, emulsions, and hydrogels have been designed to transport therapeutics for arthritis treatment [5–10]. Nanoparticles are primarily used and often administrated by intravenous injection, but the drug concentrations in plasma are unstable, and the circulation time is short. The combination systems of different carriers such as nanocomposite hydrogel composed of nanoparticle and hydrogel can overcome the single drug delivery systems limitation, increase bioavailability, enable controlled drug release, and prolong the therapeutic window, showing great potential [11–15]. Based on the characteristics of the hydrogel depot, it is usually used for subcutaneous or intra-articular delivery [16–20]. Intra-articular injection is not the best option since rheumatoid arthritis is a multi-articular inflammation. Subcutaneous administration in the back may lead to low targeting efficiency for incomplete absorption to the bloodstream and possible redistribution to lymphatic organs [21]. In Chinese medicine, stimulating the specific acupoints could regulate corresponding organs or meridians [22], and the acupoints were often near the inflammatory joints in RA treatment, so here we proposed a concept of proximal drug administration at the acupoint, which is either different from intra-articular in local or subcutaneous injection in back, which may lead to a completely different biological distribution and enhanced targeting effects. However, the basic research of drug administration in acupoints is insufficient, so this caught our interest, and the combination with nano-technology may help to image and explain the uniqueness of acupoints.

A few studies have reported that drug administration at acupoints can result in different pharmacokinetics and biological distribution [23–25], but no studies have been conducted on acupoint combination with nanocomposite hydrogel. Hence, we tried to construct an acupoint nanocomposite hydrogel and fully use the advantages of acupoint on nanocarriers and transporting drugs by a well-designed drug delivery system. Recent studies have shown the great potential of some Chinese Herbs as alternative antirheumatic drugs [26]. Triptolide (TP) is a pharmacologically active compound isolated from the Chinese herb *Tripterygium wilfordii Hook F*, which has good anti-inflammatory and immunosuppressive activities [27]. However, it has not been fully harnessed in the clinic due to its multiorgan toxicity and poor solubility [28]. The combination of drugs with nanocarriers and well-controlled hydrogel may be a solution to perform targeted and sustained delivery of proper amounts of drugs against side effects. Furthermore, it might be interesting to use this strategy of nanoparticles in gel to study the distribution and release behavior of TP in vivo after acupoint injection.

Coincidentally, hydrogel acupoint embedding may have a similar effect to acupuncture, and there have been some studies on the combination of acupuncture and hydrogel [29, 30]. Acupuncture is now a more and more popular complementary therapy for RA patients to treat pain [31]. Inspired by this, we hypothesized that it would be possible to use hydrogel to simulate acupuncture and explore the maximum potential of acupoint drug administration but not only transport anti-inflammatory agents. Based on this, we further proposed a concept of “long time acupuncture”, which exploits the molecular mechanism involved in acupuncture's antinociceptive property but does not require acupuncture needle stimulation. We realized this by controlling the release of drugs from hydrogel embedded in acupoints. Hence, we try to combine the hydrogel with acupoint and finally create an acupoint drug delivery system that integrates acupuncture’s analgesic function and explores drug delivery ability. According to literature, localized adenosine A_1_ receptor (A_1_R) plays an essential role in analgesia [32], 2-chloro-N(6)-cyclopentyl adenosine (CCPA), a selective A_1_ receptor agonist, could play the similar analgesic effect of acupuncture when it is locally administrated in the Zusanli point (ST36) [33], so we used hydrogel to load CCPA to achieve robust and long-term analgesic effect through the controlled release of CCPA.

**Fig. 1:**
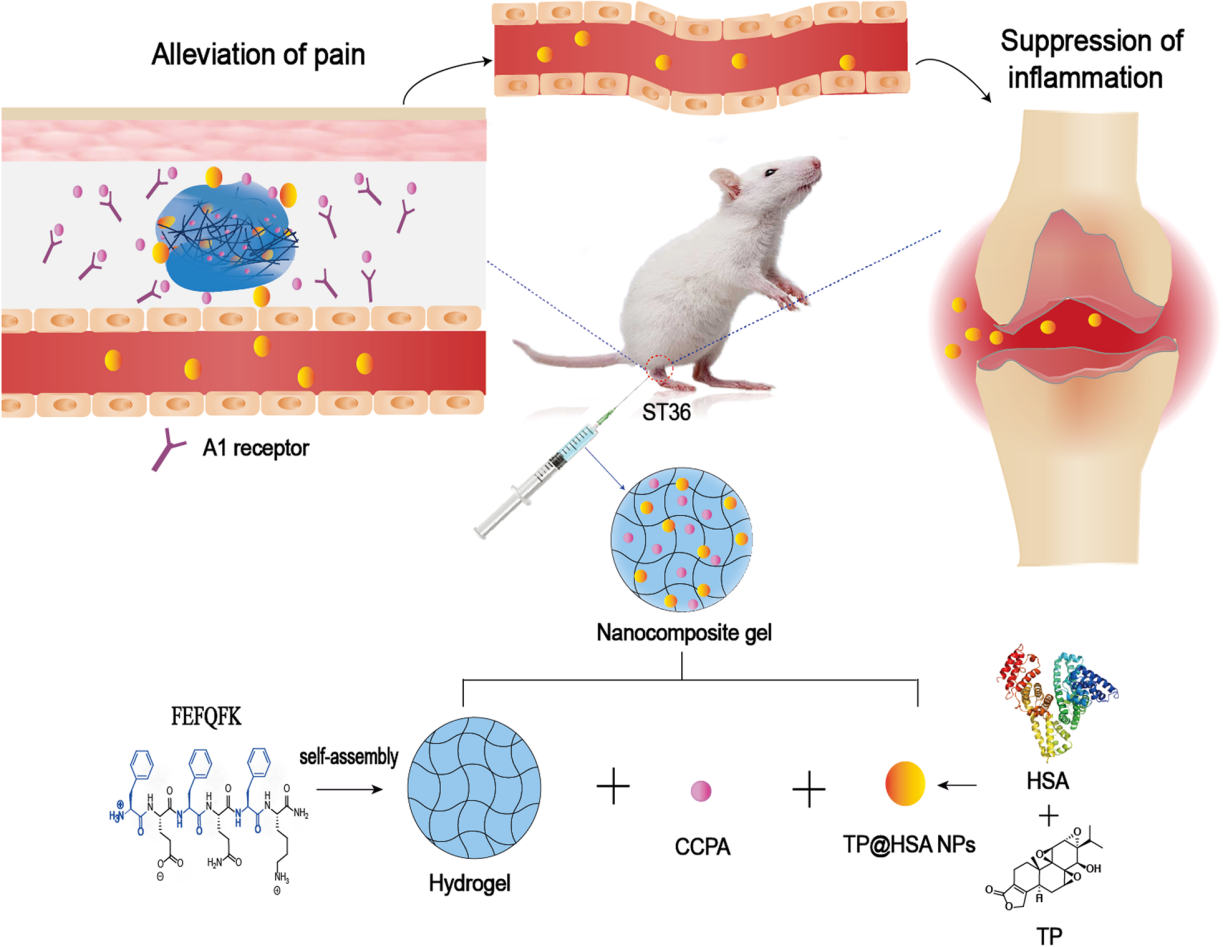
Therapeutic mechanism of nanocomposite hydrogel against RA. Acupoint nanocomposite hydrogel consists of CCPA and TP@HSA NPs not only simulate acupuncture in relieving pain by stimulating A_1_R, but also targeted deliver TP to suppress inflammation through regulating inflammatory factors and restoring immune balance

Taken together, we designed an ST36 acupoint nanocomposite hydrogel drug delivery system that can specifically treat pain and inflammation (Fig. 1). We first prepared an injectable peptide hydrogel as a depot. Then, in order to overcome the toxicity and poor solubility problem of TP, we chose human serum album (HSA), which has been proved to have a potent targeting ability to arthritic joints as a nanocarrier [34–36] to encapsulate and deliver TP (TP@HSA NPs). Finally, we used the hydrogel depot to load CCPA, which can relieve pain by stimulating acupuncture and TP@HSA NPs to perform nanocomposite hydrogel (TP@HSA NPs-CCPA-Gel) administrated it at ST36 to achieve prolonged and controlled release of the therapeutic agents. Using the adjuvant-induced arthritis (AIA) rat model, we investigated the analgesic and anti-inflammatory efficacy of our ST36 acupoint nanocomposite hydrogel. It has not been reported yet whether acupoint drug administration can change the transport behavior of drugs in vivo and benefit the treatment of RA. So, we further explored the influence of acupoint administration on a drug's biological distribution behavior. Our results demonstrated that ST36 nanocomposite hydrogel leads to specific joint accumulation, potent relief of pain, and substantial reduction of joint inflammation without increased side effects.

## Results

### Characterization of TP@HSA NPs and peptide hydrogel

**Fig. 2:**
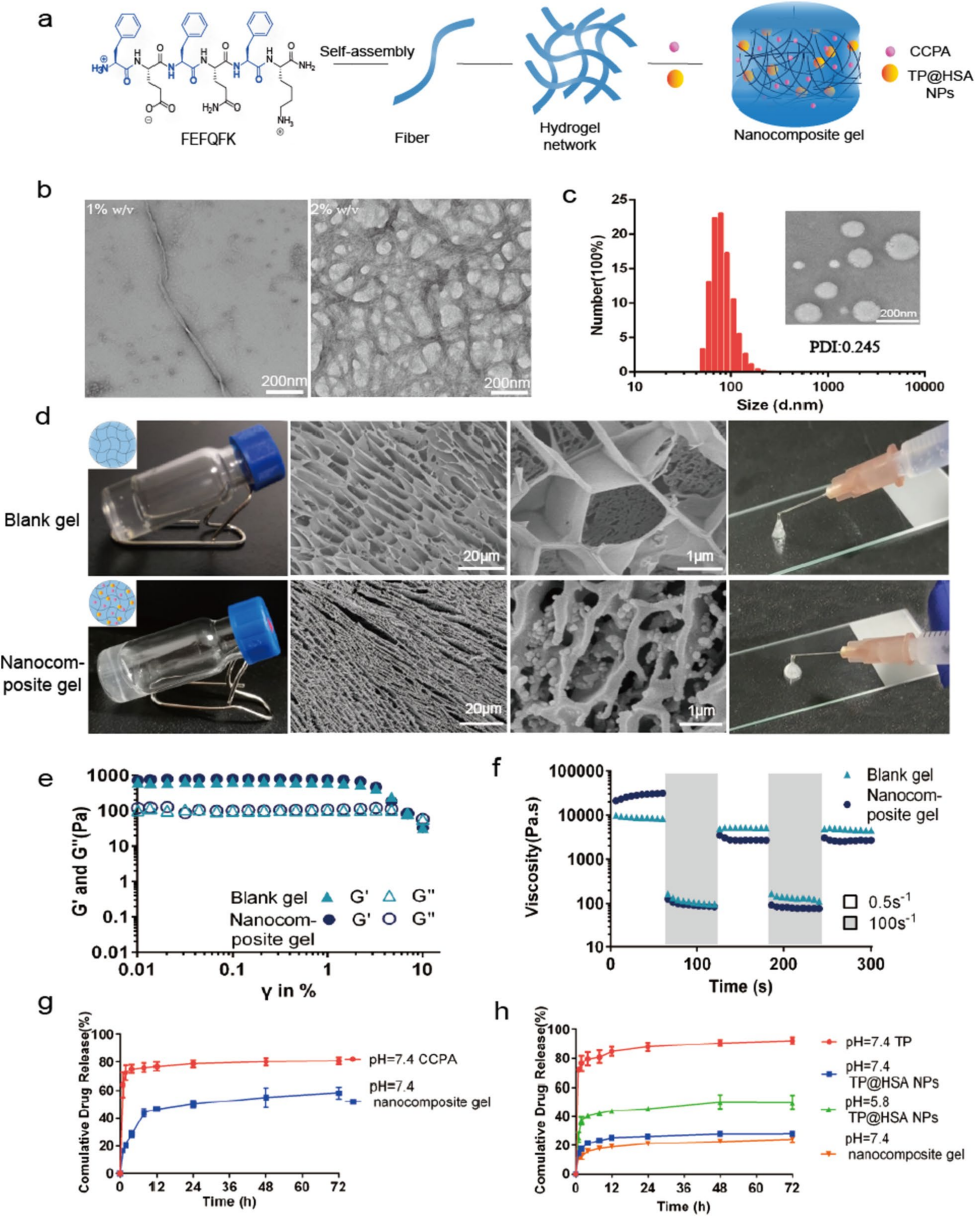
Fabrication and characterization of nanocomposite hydrogel. **a** Schematic representation of construction of nanocomposite hydrogel. **b** TEM of gel in different concentration (1% and 2%, w/v) **c** The size distribution and TEM image of TP@HSA NPs. **d** Macroscopic images,SEM and images of injection through needle showing of blank gel and nanocomposite hydrogel. **e** Strain-dependent oscillatory shear rheology of blank gel and nanocomposite hydrogel. **f** Step-shear measurements of blank gel and nanocomposite hydrogel over two cycles with alternating high shear (100 s^−1^) and low shear (0.05 s^−1^) rates. **g** Release profile of CCPA from nanocomposite hydrogel. **h** Release profile of TP from TP@HSA NPs in different pH and nanocomposite hydrogel

Herein, we prepared TP@HSA NPs following a developed method [37] and then dispersed them in the hydrogel scaffold. TEM (Fig. 2c) and SEM (Additional file 1: Fig. S2) images showed that the TP@HSA NPs were spheroidal in shape and had a uniform size. Dynamic light scattering (DLS) analysis showed that the TP@HSA NPs were well dispersed in phosphate buffered saline (PBS) (0.01 M, pH 7.4) with a hydrodynamic diameter (HD) of 91.2 ± 6.9 nm, which is in consistent with TEM and SEM. PDI is 0.245 ± 0.009 and average zeta potential was − 22.3 ± 0.6 mV. The presence of TP in the TP@HSA NPs was validated by the characteristic absorption peak of TP at 1768 cm^−1^ in the infrared (IR) spectrometer of the nanoparticles. The absorbance in 1020 cm^−1^ is enhanced without the appearance of new peak, which indicates that there is no covalent connection between TP and HSA (Additional file 1: Fig. S3). Drug loading (DL) and Encapsulation efficiency (EE) of TP in TP@HSA NPs was determined as 6.19% and 68.21%, respectively. Stability study shows that the size of particles changes slightly over 7 days, which means that nanoparticles have good stability (Additional file 1: Fig. S4).

Then, we chose an amphipathic self-assembling peptide FEFQFK to form hydrogel for its favorable biocompatibility and biodegradation which is confirmed to be a potent depot to control the release of different therapeutic agents [38, 39]. The mass of peptide is 844.5 Da and the purity is 94.85% (Additional file 1: Fig. S1). TEM images showed that there occurred some twisted ribbon morphologies in a low concentration (1%, w/v), while there came to be network when the concentration increased to 2%(w/v) (Fig. 2b).

### Fabrication and characterization of nanocomposite hydrogel

Herein, we described an acupoint hydrogel depot which can co-deliver CCPA and TP@HSA NPs (Fig. 2a). After a single injection and subsequent sol–gel phase transition, the hydrogels are localized as a “drug-release depot”, from where the released CCPA can locally activate A_1_R to exert analgesic effects, the released TP@HSA NPs can enter the lymphatic and blood circulatory system, and then effectively target the arthritis joints via HSA’s targeting ability to suppress inflammation. The morphology characterization of nanocomposite hydrogel by SEM showed that the spherical NPs were loaded in the hydrogel with network structure and the structure is tighter than blank gel (Fig. 2d). Then, we further evaluated the effect of TP@HSA NPs on mechanical properties of the gel. Strain-dependent oscillatory rheology was performed in order to quantify the strength of hydrogel before and after the addition of NPs. The results showed that blank gel and nanocomposite hydrogel displayed an extremely broad linear viscoelastic region in addition to network failure at high strains, indicating a wide processing regime and shear-thinning behavior (Fig. 2e). There is only a slight difference on elastic modulus and viscous modulus between blank gel and nanocomposite hydrogel, which indicates that nanocomposite hydrogel still remains solid-like under low stresses (i.e., before and after injection) and the addition of NPs didn’t change the rheological properties of the hydrogel. Therefore it can serve as a depot and maintain a solid gel structure after injection, which facilitates the construction of acupoint drug delivery system and preventing flow from the injection site. Injectability was tested by measuring the viscosity changes of the gels when it is under a high shear rate (100 s^−1^) of the injection process and a low shear rate (0.5 s^−1^) of the working conditions upon implantation [40]. The viscosity of both gel formulations decreased by over two cycles of magnitude with the applied high shear rate and rapidly recovered (< 5 s) to their original viscosity when returned to a low shear rate (Fig. 2f). Nanocomposite hydrogel presented higher viscosity than blank gel initially, which may be due to inter-particle interactions [41], the non-newtonian rate range is extended to low shear rates and the zero shear viscosity increases dramatically, but the recovery property is decreased due to the addition of TP@HSA NPs. Next, we evaluated the in vitro drug release behavior of CCPA and TP in nanocomposite hydrogel. CCAP was released locally at the acupoint and TP was released at arthritis joints owing to the targeting ability of HSA. As is shown in Fig. 2g, hydrogel can slow down the release rate of CCPA and the amount is 58.18% at 72 h, which is helpful for long-term analgesia. TP was released from the NPs at a higher rate in acidic conditions (pH = 5.8) than in neutral conditions (pH = 7.4) with no significant burst effect. This phenomenon was due to the changes in the conformation of HSA induced by the acidic environment. These changes altered the interactions between HSA and TP and enabled the release of the drug from the NPs. This will benefit the targeted delivery of TP to arthritis joints, whose environment is acidic. Besides, the cumulative drug release of CCPA and TP from nanocomposite hydrogel (pH = 5.8) was also evaluated. The result showed that the change of pH had little effect on TP from nanocomposite hydrogel but the cumulative release of CCPA was decreased from 58.18% at pH 7.4 to 36.17% at pH 5.8 (Additional file 1: Fig. S5). So the environment of acupoint (pH 7.4) was suitable for the release of CCPA. Besides, gel won’t cause damage to NPs and can slow down the release of TP which comes out that the cumulative release of TP is 24.29% at 72 h (Fig. 2h). This nanocomposite hydrogel delivery system is suitable for sustained and controlled drug release and can reduce the frequency of drug delivery, which will facilitate RA treatment.

Finally, the skin irritation study was performed to check the safety of the prepared hydrogel for acupoint application. The result ensured that nanocomposite hydrogel was safe and it did not cause any localized skin irritation (Additional file 1: Fig. S6).

### In vivo biodistribution of TP@HSA NPs

**Fig. 3:**
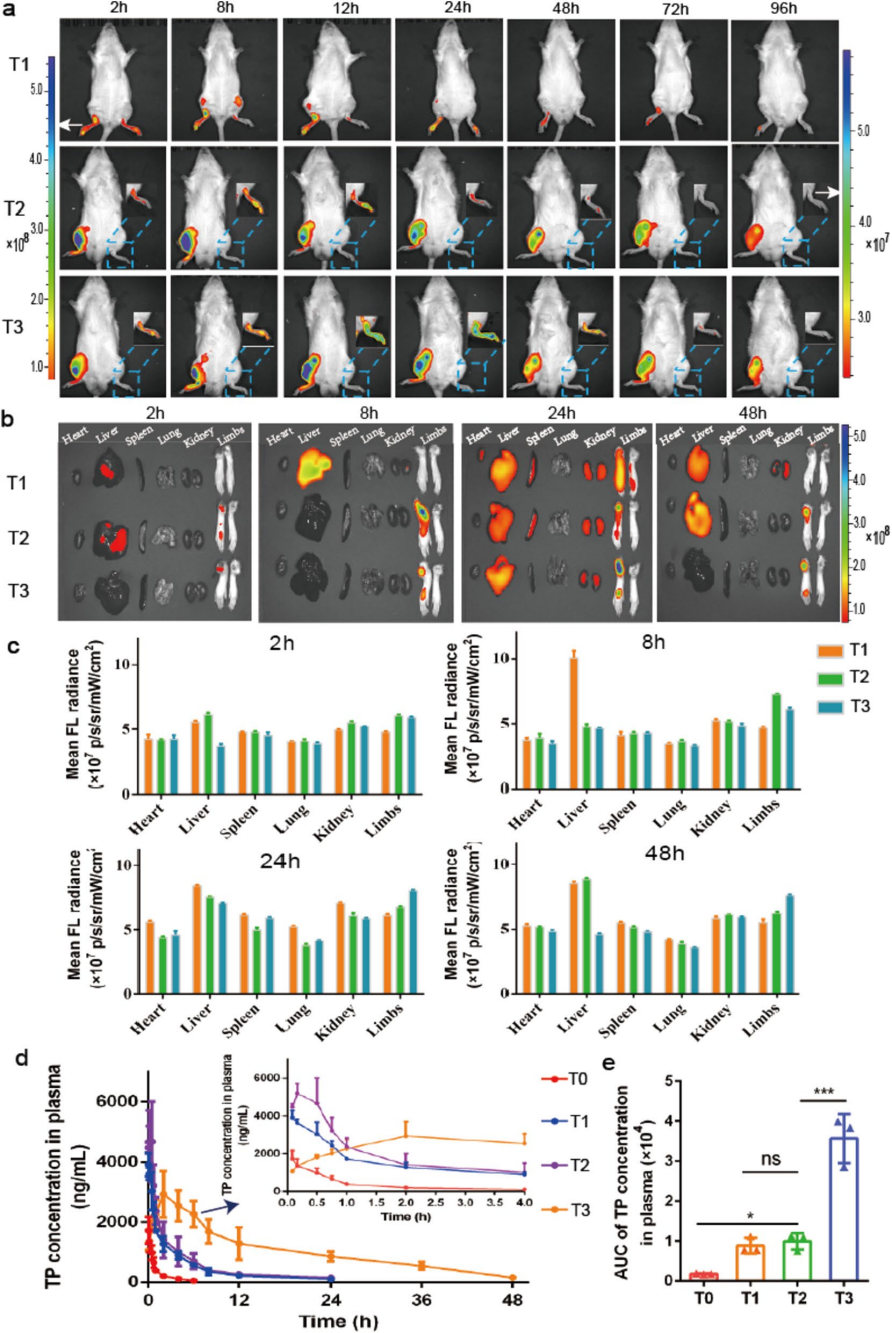
Biodistribution of TP@HSA NPs and TP@HSA NPs-Gel administrated in ST36 and non-acupoint. **a** Imaging of AIA mice treated with different preparations at different point-in-times (the fluorescence scale bar of hind paw in each side corresponds to the white arrow). **b** Imaging of organs in different groups. **c** Quantitative analysis of fluorescence in different organs at different time. **d** Pharmacokinetics of TP in different groups (the insert is local magnification of 0–4 h). **e** AUC of TP in different groups. T0: ST36 TP solution; T1: non-acupoint TP@HSA NPs; T2: ST36 TP@HSA NPs; T3: ST36 TP@HSA NPs-Gel

The combination of nanocomposite hydrogel and acupoint injection is a pioneering attempt in this study. We are interested in that whether acupoint injection can influence the transport behavior of TP@HSA NPs. Therefore, we determined the distribution of TP@HSA NPs at different time by in vivo imaging system in order to explore the difference between acupoint injection and non-acupoint injection and further to illustrate the advantages of our designed acupoint delivery system. Besides, the targeting ability of TP@HSA NPs to inflammatory joints and the property of residence and degradation of nanocomposite hydrogel at acupoints can also be monitored. The result showed that TP@HSA NPs had the ability to target the inflamed joints, which shows that the presence of fluorescence in the joints after administration gradually increased and the injection site decreased (Fig. 3a). Interestingly, we found that when TP@HSA NPs was administrated in ST36 at the other hind paw of mice, it will primely reach to the same site of joints (Additional file 1: Fig. S7), which means that ST36 administration at both hind paws can preferentially target the same site of joints. As was shown in Fig. 3b, comparing acupoint injection with non-acupoint injection, acupoint injection can better reach arthritis joints and the fluorescence intensity in liver is much less. At the first 8 h, TP@HSA NPs group could better target arthritis joints, but TP@HSA NPs-Gel group showed advantage at 24 h and 48 h, which means that gel can control the release of TP@HSA NPs and help them to slowly target arthritis joints. The fluorescence intensity in the paws of AIA rats increased over time, its maximum level occurred at 12 h in TP NPs group and 24 h in TP NPs-Gel group which means that gel can extend the release time (Fig. 3c). The effective arthritis joints targeting ability may benefit better therapeutic effect and the low toxicity of TP in the treatment for RA.

### Pharmacokinetics

Pharmacokinetic test was carried out to further validate the effect of acupoint injection on TP. The concentration of TP in circulation changed overtime in different groups was shown in Fig. 3d. Little TP was detected in the plasma of animals treated with free TP at 12 h after injection, whereas appreciable TP remained in the plasma of animals treated with ST36 TP@HSA NPs-Gel group, even at 36 h after injection. Pharmacokinetic analysis showed that AUC_0→t_ for nanocomposite hydrogel was 20.2-fold higher and 3.6-fold higher than that for free TP and TP@HSA NPs, while T_1/2_ was 4.4-fold higher and 1.5-fold higher (Fig. 3e, Additional file 1: Table S1). These results further indicate the relatively long persistence of TP in plasma in TP@HSA NPs-Gel group. However, the difference between acupoint and non-acupoint is slight (p > 0.05), maybe TP@HSA NPs all enter into blood and lymph circulation in both acupoint and non-acupoint group, so there is only a slight effect on the concentration of TP in plasma.

### Acupoint nanocomposite hydrogel can relieve pain and inhibit the development of arthritis

**Fig. 4:**
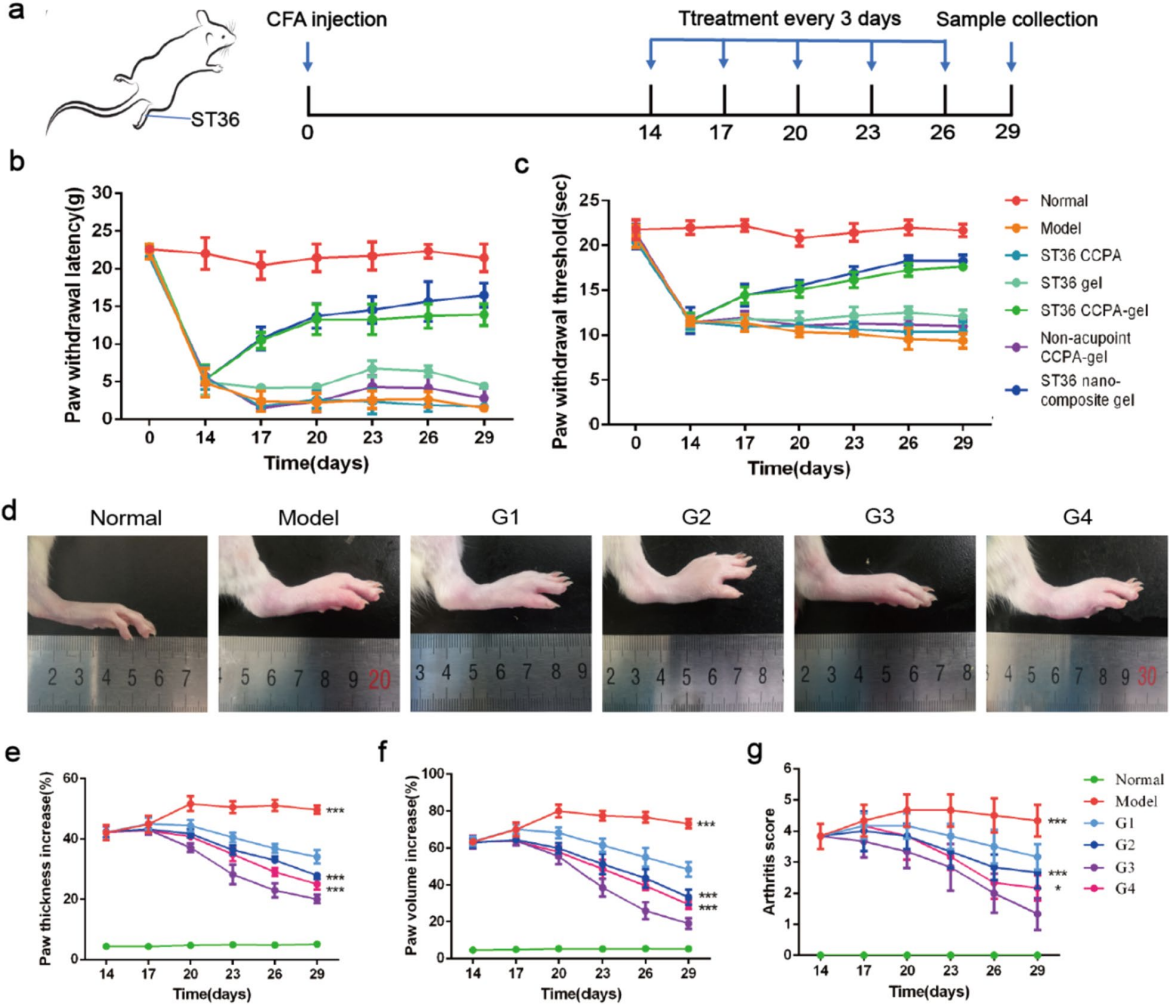
Analgesic and anti-inflammatory effects of ST36 nanocomposite hydrogel. **a** The schematic diagram of experiment procedure. **b** Mechanical allodynia (paw withdrawal latency in grams) among the different groups. **c** Thermal hyperalgesia (paw withdrawal threshold in seconds) among four groups. **d** Photographs of representative mice hand paws among the six groups. (Normal: healthy mice treated with saline, Model: AIA mice treated with saline, G1: ST36 TP-CCPA-solution, G2: ST36 TP-CCPA-Gel, G3: ST36 TP@HSA NPs-CCPA-Gel, G4: Non-acupoint TP@HSA NPs-CCPA-Gel) **e–g** Hind paw thickness, volume, and arthritis scores changes of the mice. Data (means ± SD) shown are pooled from two independent experiments with similar results. (n = 6, *p < 0.05, **p < 0.01, and ***p < 0.001)

14 days after modeling, we start the treatment every 3 days according to the schedule (Fig. 4a). It’s reported that CCPA could simulate acupuncture in relieving pain and we here loaded it in hydrogel in order to further extend the action time. So, next we checked whether CCPA-loaded hydrogel can stimulate acupuncture point to relieve pain and extend the time by both mechanical allodynia and thermal hyperalgesia experiments. As for mechanical allodynia, the paw withdrawal threshold was almost the same for all groups at day 0. After 14 days immunization of CFA, paw withdrawal latency of all groups was reduced significantly and then different treatment started (Fig. 4b). Throughout the experiment, the normal group didn’t cause any significant change in paw withdrawal latency. However, the paw withdrawal latency of negative control group was gradually reduced from 23.11 to 2.65 g. Conversely, after treatment, compared with the negative control group, ST36 CCPA-gel group exhibited little reduction in paw withdrawal from 23.43 to 13.70 g and significant improvement in the pain sensation toward touch stimuli. But the ST36 free CCPA and blank gel treated groups exhibited almost the same as the negative control group, which may ascribe to the transient effect of CCPA or hydrogel alone won’t have analgesic action. Interestingly, non-acupoint CCPA-gel didn’t show such effect, which indicated that CCPA can specially act at the acupoint. After the combination of TP@HSA NPs, nanocomposite hydrogel shows better effect than CCPA-gel group, but there is no significant difference. In thermal hyperalgesia, the normal group showed an average value of 21.77 s throughout the study. However, on the 14th day, the threshold of all other groups exhibited a significant reduction due to the development of RA. After the treatment, an increment in this threshold was observed among the different groups. The highest increment was observed for the CCPA-gel treated group (from 11.53 to 18.03 s) (Fig. 4c). These results were in accordance with the findings of mechanical allodynia, which together confirmed the specific analgesic effect of CCPA-gel in acupoint.

Previously studies have shown the therapeutic potential of TP in animal models of RA [42]. After 14 days modeling, thirty mice were randomly divided into five groups (n = 6 in each group) and the administered dose of TP was 1 mg/kg. As expected, the AIA mice treated with saline developed serious inflammation in the joints (Fig. 4d). In contrast, the mice treated with TP showed markedly lower clinical scores, group G3 was the most potent in the inhibition of joint inflammation (Fig. 4g, p < 0.001). Consistent with clinical scores, the paw thickness and volume were highest in mice treated with saline, and group G3 was the lowest. The result indicates that nanocomposite hydrogel has excellent ability in inhibiting the development of arthritis (Fig. 4e and f).

### Acupoint nanocomposite hydrogel can improve joint inflammation and delay disease progression

**Fig. 5:**
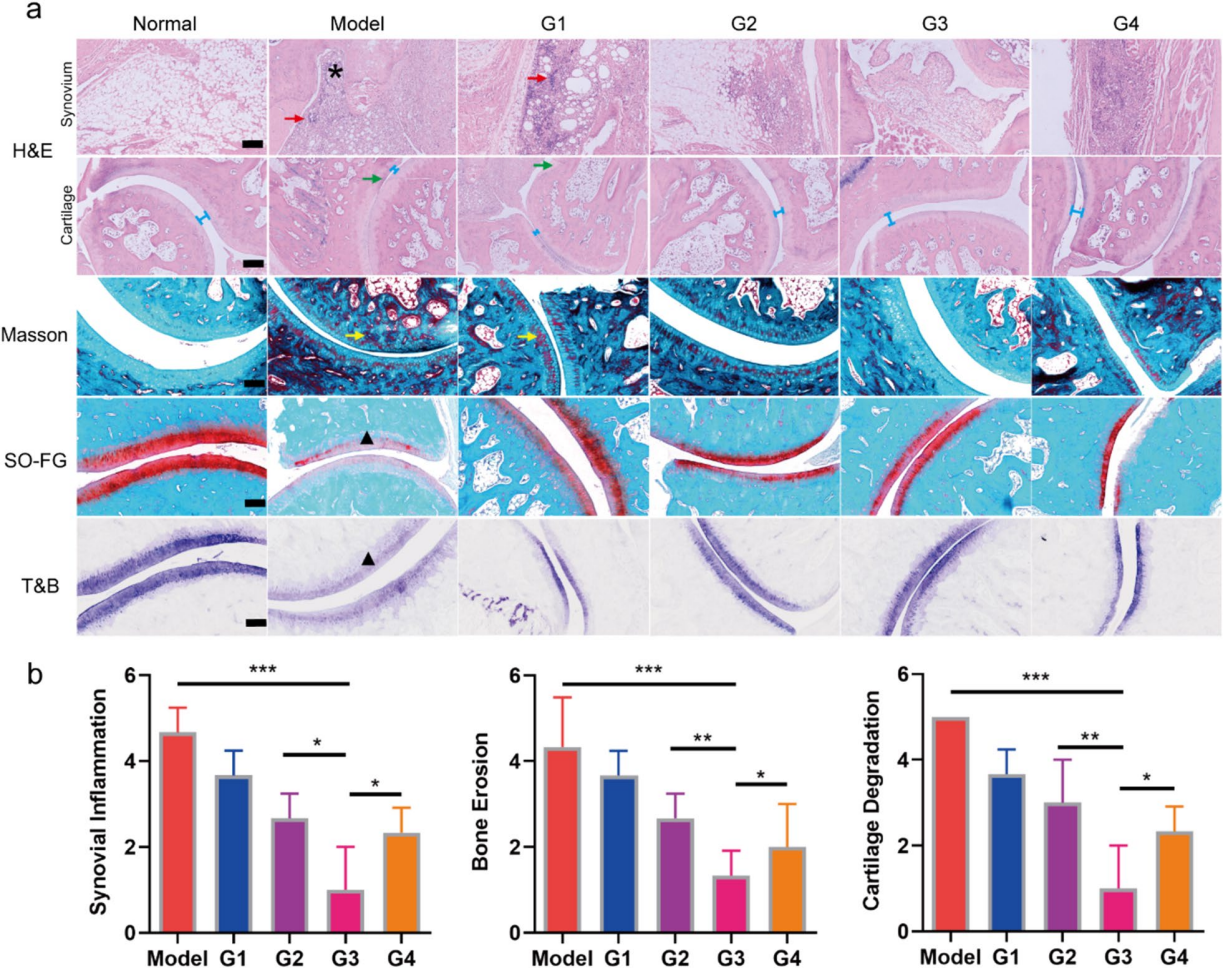
In vivo anti-arthritis efficacy of acupoint nanocomposite hydrogel. a Histological sections with H&E, Masson, SO-FG and T&B staining of joints in different groups. G1: ST36 TP-CCPA-solution, G2: ST36 TP-CCPA-Gel, G3: ST36 TP@HSA NPs-CCPA-Gel, G4: Non-acupoint TP@HSA NPs-CCPA-Gel)**.** Asterisks: formation of pannus. Red arrows: invasion of inflammation. Green arrows: destruction of cartilage. Blue line: narrow joint space. Yellow arrows: destruction of cartilage. Black triangle: loss of proteoglycan. Scale bar: 200 μm. b Histopathologic scores of synovial inflammation, bone erosion, and cartilage degradation. *p < 0.05, **p < 0.01, ***p < 0.001

RA is characterized by sustained synovitis, progressive cartilage and bone destruction [43]. To validate pathological states of bone, cartilage, and synovium at the end of treatment, histological analysis of the ankle joint sections was performed. Hematoxylin and eosin (H&E) staining demonstrated extensive inflammatory cells infiltration (red arrows) in the articular cavity, cartilage destruction (green arrows) and obvious formation of pannus (asterisks) in inflamed joints of rats in model group (Fig. 5a). In contrast, group G3 showed near normal articular cavity surfaces with a clear interface and minimal cells infiltration. Masson staining was also carried out and the result is in consistent with H&E. In addition, Safranin O-fast green (SO-FG) staining showed that obvious proteoglycan loss was found in model group, indicating serious damage in the cartilage. Again, group G3 exhibited the best cartilage preservation, comparable to the normal group (Fig. 5a). Toluidine blue (T&B) staining in the model group exhibited reduced coloration (triangle) in the cartilage when compared with the normal group (Fig. 5a), implying that the glycosaminoglycans were largely lost. After intervention with acupoint nanocomposite hydrogel (group G3), the coloration was darker than that of the other three groups, which means the best protective effect of group G3. The result of histopathology further confirmed the therapeutic effect of acupoint nanocomposite hydrogel in anti-inflammation and delaying disease progression (Fig. 5b). This result is in consistent with micro-CT, which shows that the negative control group exhibited a higher degree of bone erosion while group G3 only shows slight erosion (Additional file 1: Fig. S8).

**Fig. 6:**
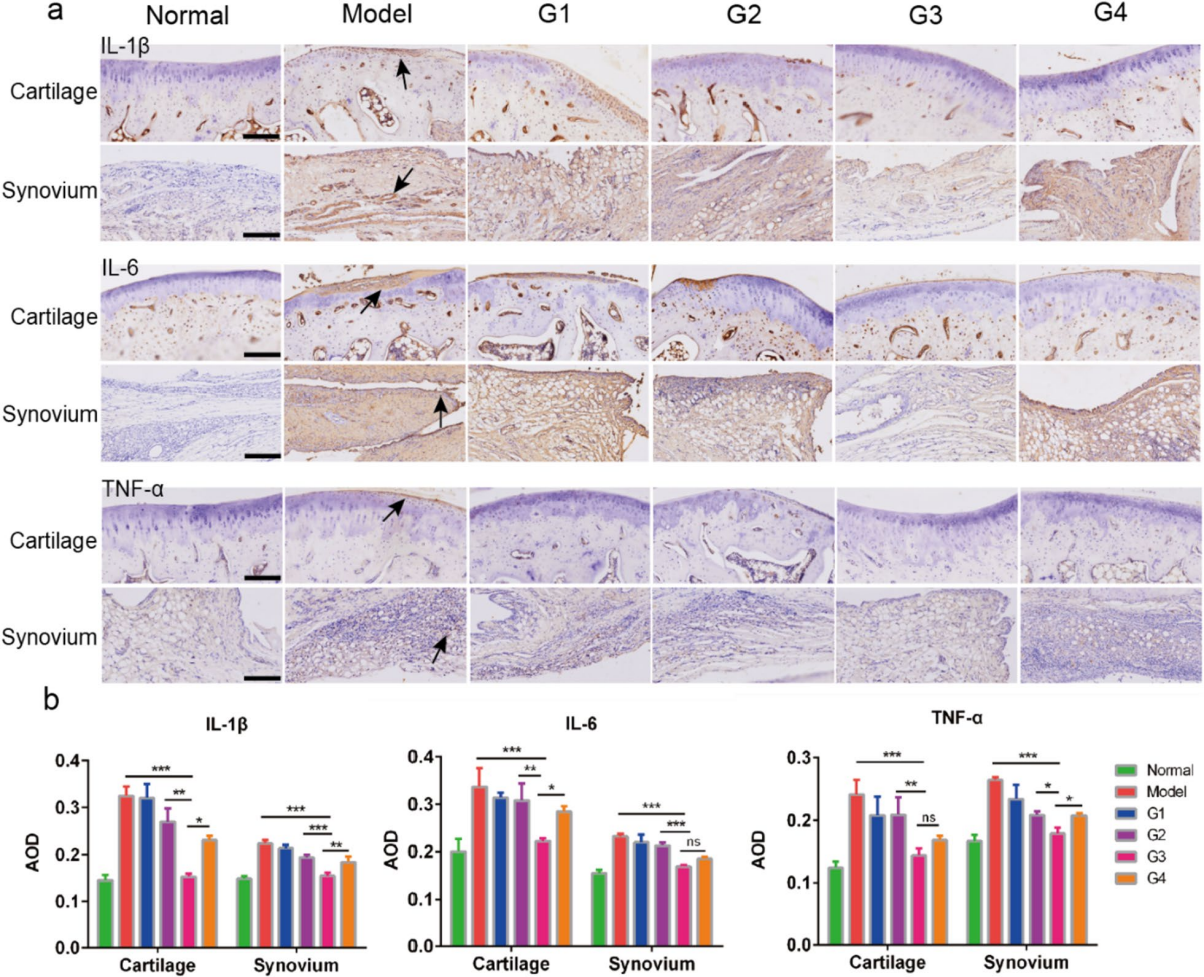
Immunohistochemistry analysis of articular tissues. **a** immunohistochemical image of cartilage and synovium in different groups. Black arrows: positive staining. Scale bar:100 μm. **b** Quantification of the IL-1β, IL-6 and TNF-α production in the tissues treated with different samples based on the immunohistochemical results. G1: ST36 TP-CCPA-solution, G2: ST36 TP-CCPA-Gel, G3: ST36 TP@HSA NPs-CCPA-Gel, G4: Non-acupoint TP@HSA NPs-CCPA-Gel**.** Data are reported as mean ± SD (n = 3), *p < 0.05, **p < 0.01, ***p < 0.001

As previously reported, the proinflammatory cytokines TNF-α, IL-1β and IL-6 might be potential therapeutic targets in RA [44]. In addition, TP is an anti-inflammatory drug that can suppress these cytokines [45]. Thus, the anti-inflammatory effect of the TP based nanocomposite hydrogel was further evaluated and the mechanism was investigated through the histological examination of proinflammatory cytokine expression [10]. As shown in Fig. 6a and b, the expression of TNF-α, IL-1β and IL-6 in the saline group was significantly elevated compared with the healthy group, indicating the central involvement of these cytokines in the pathogenesis of RA. After treatment, the expression of these cytokines decreased and group G3 showed the best result compared with the other three. The result of proinflammatory cytokine expression is in accordance with the reduced synovial inflammation and cartilage erosion observed.

### Acupoint nanocomposite hydrogel regulates inflammatory cytokine production and restores the balance of Th17 and Treg cells

**Fig. 7:**
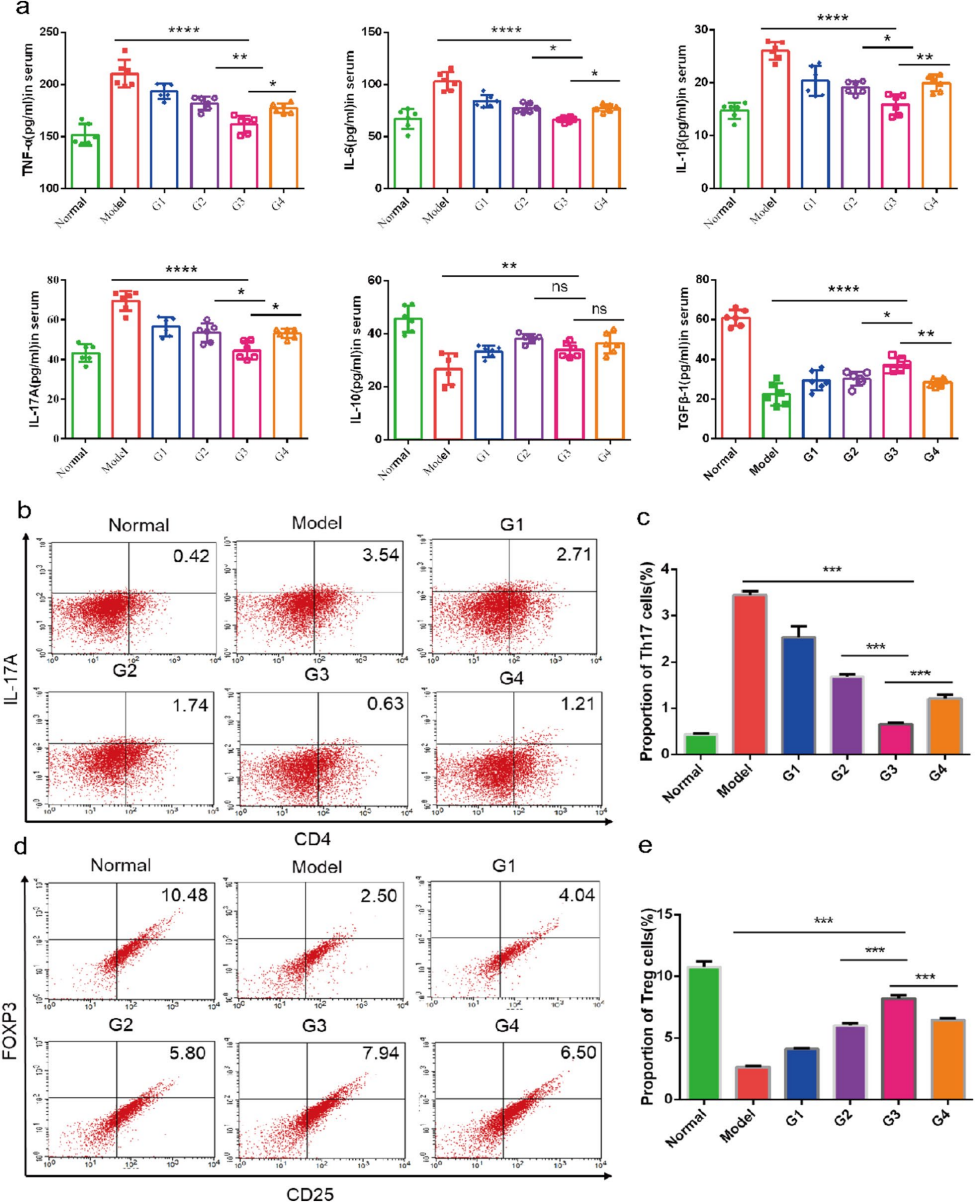
Evaluation of regulating the expression of inflammatory factors and immune cells.** a** Serum concentration of pro-inflammatory cytokines (TNF-α, IL-1β, IL-6 and IL-17A) and anti-inflammatory cytokines (IL-10 and TGF-β1). **b**–**e** FACS data is shown on the left panel. The right panel shows the summarized data. G1: ST36 TP-CCPA-solution, G2: ST36 TP-CCPA-Gel, G3: ST36 TP@HSA NPs-CCPA-Gel, G4: Non-acupoint TP@HSA NPs-CCPA-Gel. Data are reported as the mean ± SD (n = 6, *p < 0.05, **p < 0.01, and ***p < 0.001)

The pathogenesis of RA is still unclear. New therapeutic strategies that aim to achieve immune homeostasis in the joint by balancing inflammation and inducing its resolution has great potential. The TP based treatment group showed a significant reduction in pathology change in comparison with the untreated groups. Our object is to balance and resolve, rather than just suppress inflammation. Hence, we further examined the effect of different treatment groups on the expression of both pro-inflammatory and anti-inflammatory cytokines in AIA mice. TNF-a, IL-6, IL-1β, IL-17A, IL-10 and TGF-β1 were measured with ELISA kits. Our results indicated that the treatment suppressed the pro-inflammatory cytokines (TNF-α, IL-6, IL-1β and IL-17A) but up-regulated the anti-inflammatory cytokines (TGF-β1 and IL-10), indicating that the therapeutic effect of TP in RA may simultaneously target various cytokines. Among all the treatment groups, G3 is the best (Fig. 7a), which means that acupoint nanocomposite hydrogel has synergistic anti-inflammatory potential. On one side, it maybe because the gel stimulates acupoint production and affects the release of substances from local tissues. On the other side, acupoint injection allows the drug to reach more to the inflamed joint and has better treatment effects.

Building on the result of ELISA that Th17 cell-related cytokine (IL-17A) decreased, while Treg cell-related cytokines (IL-10 and TGF-β1) increased. We further tested the ability of acupoint nanocomposite hydrogel to re-establish the balance of Th17/Treg. Since Th17 cells and Treg cells are two important T lymphocyte subsets with opposing actions [46]. In RA development, the proportion of Treg cells decreases and their function is inhibited [47]. The resulting imbalance of Th17/Treg may be responsible for the occurrence and development of RA. Therefore, restoration of the Th17/Treg balance may have a potential role in the treatment of RA. After treatment, the CD4 + CD25 + FOXP3 + Treg cells were markedly increased while CD4 + IL-17A + Th17 cells were markedly decreased. The result showed that acupoint nanocomposite hydrogel was better than acupoint TP-gel and non-acupoint nanocomposite hydrogel (Fig. 7b–e), which means that HSA NPs can have better treatment effect than free TP and acupoint injection may have synergistic therapeutic effect.

### Acupoint nanocomposite hydrogel can reduce multiorgan toxicity of TP

**Fig. 8:**
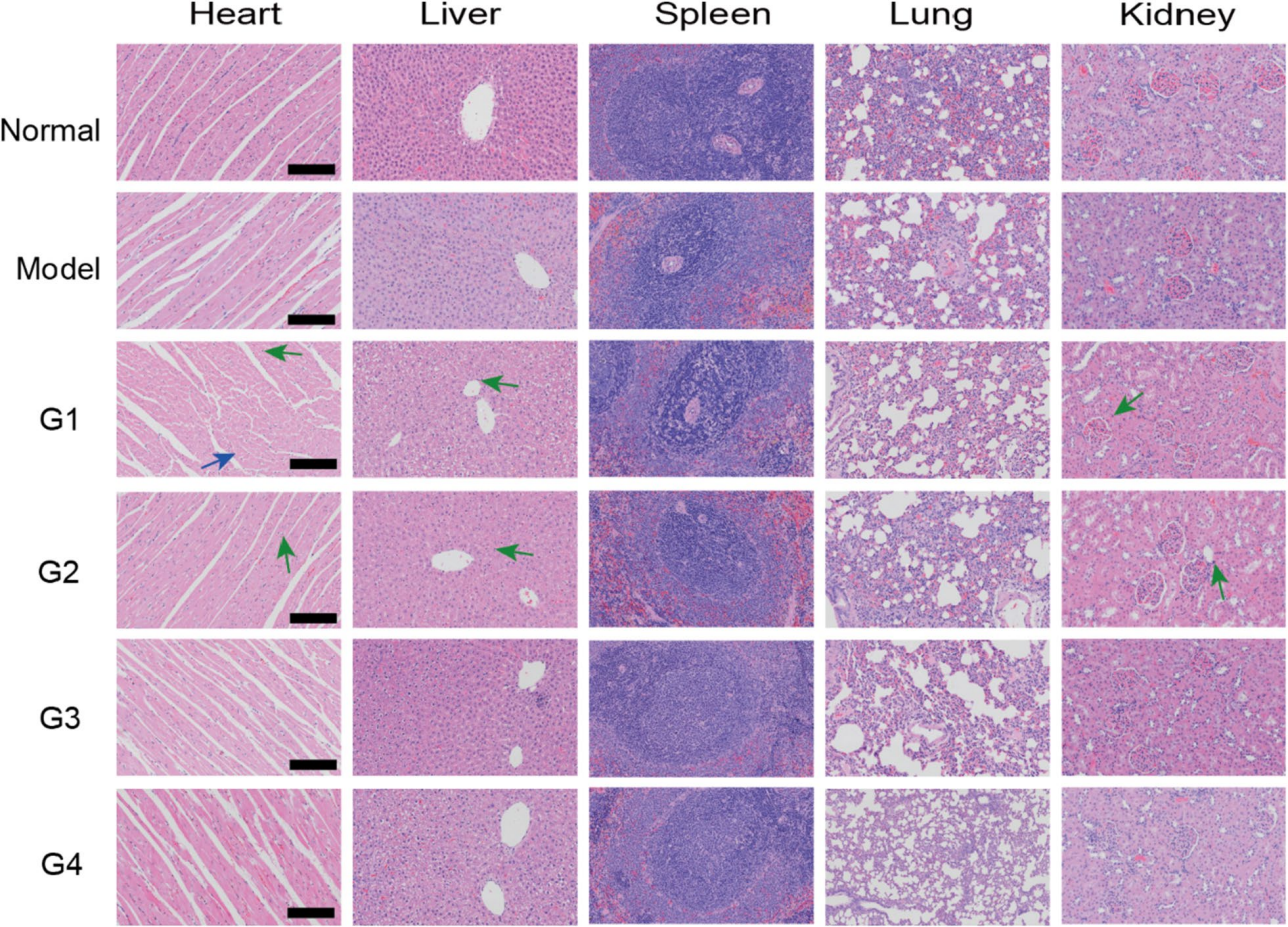
H&E-stained images of major organs (heart, liver, spleen, lungs, and kidneys), Blue arrow represents myocardial cell swelling and green arrow represents inflammatory cells infiltration. G1: ST36 TP-CCPA-solution, G2: ST36 TP-CCPA-Gel, G3: ST36 TP@HSA NPs-CCPA-Gel, G4: Non-acupoint TP@HSA NPs-CCPA-Gel**.** Scale bar:100 μm

It is well known that TP possesses severe toxic effects, such as multiorgan or tissue damage and even death [48]. We thus evaluated the safety of TP based nanocomposite hydrogel and whether acupoint injection can collectively help to reduce toxicity. We performed histopathological analysis of major organs including heart, liver, spleen, lungs, and kidneys. The result showed that free TP has severe liver and heart toxicity which supported by myocardial cell swelling, myocardial fiber atrophy*/*breakage and inflammatory cell infiltration and feathery necrosis and moderate inflammatory cell infiltration of liver tissues, respectively (Fig. 8). Group G3 treated with nanocomposite hydrogel had lower toxicity, and acupoint injection had better effect compared with non-acupoint injection. This may be attributed to less general distribution of TP.

## Discussion

In clinic, RA has to face the toxicity problem of the long-term use of analgesics and anti-inflammatory drugs. Acupuncture is a way to replace analgesics, and targeted deliver anti-inflammatory drugs was also a better choice, so an acupoint delivery system with specific analgesic and anti-inflammatory effects was constructed. This combination of nanocomposite hydrogel and acupoint provides a new idea for integrating acupuncture and drug administration. Supramolecular hydrogels made by self-assemble peptide, with good viscoelasticity, can be used as a reservoir for acupoint injection. CCPA and TP@HSA NPs were loaded in this gel and administrated in ST36 for sustained and controlled release. On one hand, the acupuncture mechanism was used locally for analgesia at the acupoints. On the other hand, TP could be delivered slowly from the acupoints to the inflamed joints. The interaction between nanoparticle and gel was evaluated by the characterization of the physical and chemical properties of the nanocomposite hydrogel system. The results showed that the addition of nanoparticles made the gel cross-linking tighter and improved the mechanical strength, while the gel further delayed the release of drugs from the nanoparticles. The results of the analgesia experiment showed that nanocomposite hydrogel acupoint administration have longtime analgesic effect. The efficacy results showed that acupoint nanocomposite hydrogel could effectively improve the swelling, reduce the arthritis score and the expression of pro-inflammatory factors in the joint. In addition to the improvement of local pathology, acupoint nanocomposite hydrogel can also regulate systemic inflammatory factors, reducing the expression of pro-inflammatory factors and increasing the expression of anti-inflammatory factors. Furthermore, it can restore the immune balance of Th17/Treg. Finally, the biosafety evaluation results showed that the acupoint nanocomposite hydrogel could significantly reduce the toxic and side effects of TP on organs, which helps to improve the safety of treatment. As shown from the skin irritation test, the delivery system is not only safe for injection, but also achieves the biosecurity for drug delivery.

## Conclusions

In this study, we innovatively developed a new delivery system combined a long-acting acupoint acupuncture with a local drug depot, which exhibits longtime analgesic effects, joint inflammation improvement, immune balance reconstruction of Th17/Treg and side effects alleviation of TP. It is a groundbreaking attempt to combine acupuncture and drugs in the treatment of RA.

## Methods

### Materials and animals

Triptolide (TP, > 98%) was purchased from feiyu biological technology company (Nantong, China), Human serum album (HSA, MW 66.5 kDa) and phosphate buffer saline (PBS, 0.01 M) were purchased from solarbio science and technology company (Beijing, China). Dimethyl sulfoxide (DMSO) and other organic solvents were purchased from Sigma-Aldrich (USA). All chemicals were reagent-grade or higher and directly used without further purification. Sprague–Dawley (SD) male rats aged 6–8 weeks and weighing 180–220 g were purchased from the Experimental Animal Center of Nanjing University of Chinese Medicine (Nanjing, China). All animal experiments were carried out following the National Institutes of Health (NIH, USA) guidelines for the care and use of laboratory animals in research. The surgical procedures and experimental protocols were approved by the Committee for Animal Experiments of Nanjing University of Chinese Medicine.

### Preparation and characterization of TP@HSA NPs and peptide

TP loaded HSA nanoparticles (TP@HSA NPs) were fabricated by a TP induced self-assembly of HSA. First, TP (20 mg/mL) was dissolved in dimethyl sulfoxide (DMSO), and HSA (5 mg/mL) was dissolved in PBS with vortex. A 50 μL amount of TP solution was added dropwise to the 2 mL of HSA solution under vigorous stirring. After that, the mixture was stirred for 3 h at room temperature in the dark. Then 2 mL of alcohol was dropped into the mixture via an automatic injection pump at the speed of 0.5 mL/min. After 5 min under stirring, organic solvent was removed by using a rotary evaporator (R300, BUCHI, Switzerland) at 40 °C. Then TP@HSA NPs were obtained after ultrafiltration by a centrifugal filter device (MWCO = 10 kDa) three times and resuspended in PBS. The particle size, polydispersity index (PDI) and δ potential of nanoparticles were determined by dynamic light scattering (DLS, Nano-Zetasizer, Malvern, UK) at 25 °C. Morphologies of TP@HSA NPs were determined by transmission electron microscope (TEM, HT7800, Hitachi, Japan) with an acceleration voltage at 100 kV. Drug loading (DL) and encapsulation efficiency (EE) were calculated by the following formulas: DL (%) = (mass of TP encapsulated in nanocarriers)/(mass of TP-based drug delivery systems) × 100%; EE (%) = (mass of TP encapsulated in nanocarriers)/(mass of TP added) × 100%.

FEFQFK peptide was synthesized using a standard FMOC based solid-phase peptide synthesis. Detailed experimental method is available in the supporting information. Crude peptides were purified by preparative reverse phase high-performance liquid chromatography (prep-HPLC, PRE150Q, Waters, USA). The resulting pure peptides were obtained after lyophilisation of the collected fractions. The mass of peptide was confirmed by mass spectroscopy using LC/MS (1290II-6460, Agilent, USA) and its purity was confirmed by HPLC (LC-2010A HT, SHIMADZU, Japan). Morphology of hydrogel was determined by transmission electron microscope (TEM, HT7800, Hitachi, Japan) with an acceleration voltage at 100 kV.

### Preparation and characterization of nanocomposite hydrogel

A predetermined amount of CCPA was dissolved in DMSO and TP@HSA NPs were added into a peptide hydrogel (w/v: 2%) to form TP@HSA NPs-CCPA-Gel, and thus a homogeneous and free flowing injectable hydrogel loading TP@HSA NPs and CCPA was obtained. The morphology of this nanocomposite scaffold was characterized by SEM (JEOL 7600F with Gatan Alto). The mechanical strength was evaluated by rheology (MCR302, Anton Paar, Germany).

### In vitro release of CCPA and TP

To evaluate the in vitro release behavior of CCPA and TP in nanocomposite hydrogel, 1 mL nanocomposite hydrogel was added into a dialysis bag (MW 1000) and immersed in 20 mL of PBS with 0.5% (v/v) Tween 80 at 37 °C. Then, PBS was collected and the equal volume of fresh PBS was added over an indicated interval. Besides, in order to mimic the drug release from TP@HSA NPs in vivo, release medium with different pH value was used to check the release pattern of TP to correlate with physiological pH (blood pH 7.4) and inflammatory sites (endosome and lysosome pH, i.e., 5) [49]. The amount of cumulative CCPA and TP was then measured by HPLC (2010AT, Shimadzu, Japan) with a UV–Vis detector at 218 nm. A mixture of acetonitrile/water (40/60, v/v) was used as the mobile phase. Separations were carried out on a C18 reverse phase column, the flow rate was 0.8 mL/min and the temperature was 30 °C. All experiments were repeated three times.

### Behavioral experiments

RA was induced through intradermic injection of Complete Freud’s Agent (CFA) (100 µL) into the mice footpad. Mechanical allodynia was assessed using Von Frey filaments (Stoelting, USA) according to the previously described method. Mice were placed in a clear plastic chamber on a stainless steel mesh floor and allowed to acclimatize. Von Frey filament was applied on the plantar surface of the right hind paw with gradual increase in the applied force. Paw withdrawal threshold in response to the mechanical stimulus was determined using a series of Von Frey filaments47 (Exacta, California 95020- USA). Thermal hyperalgesia was assessed using an Analgesymeter (Ugo Basile). A mobile radiant heat source was focused on the hind paw and the paw withdrawal latencies were defined as the time taken by the mouse to remove its hind paw from the heat source (maximum of 20 s to avoid tissue damage). The paw withdrawal was repeated three times for each foot and the average calculated. To avoid conditioning to stimulation, we interposed a 5-min rest period between each trial in both thermal and mechanical tests.

### In vivo biodistribution of TP@HSA NPs in arthritis rats

Cy7-NHS labeled HSA was prepared according to the described method before [50]. Briefly, 1 mg of Cy7-NHS ester was dissolved in 20 mL of PBS solution (pH 8.0) containing 100 mg of HSA and stirred at room temperature in the dark for 8 h. After the reaction, excess Cy7-NHS dye was removed by dialysis against 10 mM PBS solution using a dialysis membrane (MWCO = 3500 Da) followed by free-dying. Then we use the Cy7-NHS labeled HSA to prepare TP@HSA NPs followed by the above procedure. Finally, we injected a TP dose of 1.0 mg/kg in each mouse and imaged at predefined time points (2, 8, 12, 24, 48, 72 and 96 h) under the IVIS® Spectrum system (Caliper, Hopkington, MA, USA). Besides, in order to evaluate the distribution in different organs, the rats were sacrificed at 2, 8, 24 and 48 h. The fluorescence signals in the collected organs (heart, liver, spleen, lung, kidneys and paws) were observed by IVIS® Spectrum system.

### Pharmacokinetics of TP@HSA NPs

A total of 12 AIA male Sprague Dawley rats (180–220 g) were randomly divided into four groups (n = 3), the groups are ① ST36 TP solution, ② Non-acupoint TP@HSA NPs, ③ ST36 TP@HSA NPs, ④ ST36 TP@HSA NPs-Gel. The dose of TP was 2 mg*/*kg in each group. At the indicated time points, blood samples (0.3 mL) were drawn from the orbit using a 0.5-mm capillary tube and immediately mixed with heparin (1%, w/v). Plasma samples were extracted using methanol, and the resulting extracts were analyzed using liquid chromatography–mass spectrometry (API55000, SCIEX, USA). TP (MW 361 Da) was detected at the m/z transition 361.0 → 113.9. Pharmacokinetic parameters were calculated using DAS2.0 (Mathematical Pharmacology Professional Committee of China, Shanghai, China).

### In vivo therapeutic efficacy of ST36 nanocomposite hydrogel

AIA mice were divided in six groups (n = 6) based on the inflammation score and hind paw volume to create the most homogeneous groups in terms of inflammation. Then, they were treated every 3 days for a total of 5 times. The dose of TP is 1 mg/kg and the dose of CCPA is 100 μg/kg. The groups are ① Normal(healthy mice without arthritis induction and any treatment), ② Model(AIA mice treated with ST36 saline), ③ G1(AIA mice treated with ST36 TP-CCPA solution), ④ G2(AIA mice treated with ST36 TP-CCPA-Gel), ⑤ G3(AIA mice treated with ST36 TP@HSA NPs-CCPA-Gel), ⑥ G4(AIA mice treated with non-acupoint TP@HSA NPs-CCPA-Gel). ST36 located at 3–4 mm below and lateral 1–2 mm for the midline of the knee. Non-acupoint located at subcutaneous area in the back where is usually used for subcutaneous injection. The thickness, volume and arthritis score of hind paws were measured to evaluate the therapeutic efficacy at every 3 days. Photographs of hind paws were taken before and after different treatments.

### Histology study and immunohistochemistry

The collected limbs in each group (n = 3) were dissected from each group and fixed in 4% paraformaldehyde for 48 h, and then decalcified in 10% neutral EDTA solution for 15 days at room temperature [51]. Then decalcified tissue was embedded in paraffin and sliced for H&E, SO-FG, Masson and T&B staining according to the manufacturer’s protocol. The slices were recorded by microscopy (OLYMPUS BX43). Subsequently, the histopathologic scores of synovial inflammation, bone erosion, and cartilage degradation are evaluated (Additional file 1: Table S2).

Immune-fluorescent staining was carried out as follows. The specimens were deparaffinized, and the antigens were retrieved by microwave. The sections were blocked using 3% BSA for 30 min, followed by incubation with primary antibodies (TNF-α, IL-Iβ and IL-6) overnight at 4 °C. After washing, the sections were incubated with biotinylated secondary antibody for 50 min and subsequently incubated with streptavidin solution. Then, 10 μL of 3,3-diaminobenzidine tetrahydrochloride (DAB) was added as a chromogen, followed by counterstaining with hematoxylin. After staining, the sections were dehydrated through increasing concentrations of ethanol and xylene. The sections were then observed by microscopy (OLYMPUS BX43), and the staining intensity in each of 3 randomly selected fields per sample was calculated using ImageJ software.

### ELISA assay

Serum samples were obtained from the mice on day 29 and kept at − 80 °C until analysis. The presence of IL-6, TNF-α, IL-1β, IL-17A, IL-10 and TGF-β1 in serum was measured with ELISA kits (Mlbio, China) according to the manufacturer's instructions.

### Flow cytometry

Antibodies purchased from eBioscience consisted of anti-CD4-FITC, anti-CD25-APC and anti-Foxp3-PE antibodies. The spleens were harvested, lyzed, and single-cell suspensions were prepared. Next, the cells were labeled with anti-mouse CD4 antibodies before permeabilization with Cytoperm/Cytofix (Becton Dickinson) according to the manufacturer’s instructions. After permeabilization, the cells were incubated with labeled antibodies that were specific for either mouse IL-17 or FoxP3. Then, the cells were centrifuged and the pellets were washed to remove unbound antibodies. After surface and intracellular labeling, mononuclear cells were analyzed by FACS Calibur (Becton Dickinson, USA) according to the manufacturer's instructions.

### Safety evaluation

During the treatments, the body weight of each mouse was recorded. Then the mice were sacrificed 2 days after the treatment. Major organs including heart, liver, kidney, lung and spleen were extracted, fixed in 4% para-formaldehyde and stained with H&E.

### Statistical analysis

Statistical analysis was performed using GraphPad Prism 6.0 (GraphPad Software, La Jolla, CA, USA). Comparative analysis of the difference between groups were performed by one-way ANOVA (Dunnett's multiple comparisons test). All the values were presented as the mean ± SD. A statistically significant difference was determined at *p < 0.05.

## Supplementary Information

**Additional file 1:** **Table S1.** Pharmacokinetic of TP in different groups. **Table S2.** Histopathological scores. **Figure S1.** Molecular weight and purity verification of FEFQFK sequence. **Figure S2.** SEM of TP@HSA NPs. **Figure S3.** FTIR spectra of HSA, TP, the mixture of HSA and TP, and TP@HSA NPs. **FigureS4.** Study on the stability of TP@HSA NPs. **Figure S5.** Comparative of the release behavior of CCPA and TP from nanocomposite hydrogel in different pH. **Figure S6.** Histopathological images obtained by H&E staining. **Figure S7.** Imaging of AIA mice treated with TP@HSA NPs in ST36 in left site at different point-in-times. **Figure S8.** Representative micro-CT images of the ankle joints at day 28.

## Data Availability

The datasets generated and/or analysed during the current study are not publicly available due to their large size, but are available from the corresponding author on reasonable request.

## Abbreviations

RA: Rheumatoid arthritis
TP: Triptolide
CCPA: Cyclopentyl adenosine
TP@HSA NPs: Triptolide-human serum album nanoparticles
DDSs: Drug delivery systems
A_1_R: A_1_ receptor
HSA: Human serum album
TP@HSA NPs-CCPA-Gel: TP@HSA NPs and CCPA loaded hydrogel
AIA: Adjuvant-induced arthritis
TEM: Transmission electron microscope
SEM: Scan electron microscope
DLS: Dynamic light scattering
PDI: Polydispersity index
PBS: Phosphate buffered saline
HD: Hydrodynamic diameter
IR: Infrared spectrometer
DL: Drug loading
EE: Encapsulation efficiency
H&E: Hematoxylin and eosin
SO-FG: Safranin O-fast green
T&B: Toluidine blue
DMSO: Dimethyl sulfoxide
CFA: Complete Freud’s Agent
DAB: Diaminobenzidine tetrahydrochloride
