# Supplementary material for: Acupoint nanocomposite hydrogel for simulation of acupuncture and targeted delivery of triptolide against rheumatoid arthritis
Source: J Nanobiotechnology. 2021 Dec 7;19:409. doi: 10.1186/s12951-021-01157-z (PMC8650546; doi:10.1186/s12951-021-01157-z)
Supplement: Supplementary file 1 — Additional file 1: Table S1. Pharmacokinetic of TP in different groups. Table S2. Histopathological scores. Figure S1. Molecular weight and purity verification of FEFQFK sequence. Figure S2. SEM of TP@HSA NPs. Figure S3. FTIR spectra of HSA, TP, the mixture of HSA and TP, and TP@HSA NPs. FigureS4. Study on the stability of TP@HSA NPs. Figure S5. Comparative of the release behavior of CCPA and TP from nanocomposite hydrogel in different pH. Figure S6. Histopathological images obtained by H&E staining. Figure S7. Imaging of AIA mice treated with TP@HSA NPs in ST36 in left site at different point-in-times. Figure S8. Representative micro-CT images of the ankle joints at day 28. [file 12951_2021_1157_MOESM1_ESM.docx]

**Additional file**

**Acupoint nanocomposite hydrogel for simulation of acupuncture and targeted delivery of triptolide against rheumatoid arthritis**

Shujing Ren^1,3#^, Heng Liu^1,4#^, Xitong Wang^1^, Jiquan Bi^1^, Shengfeng Lu^2^, Chenqi Zhu^5^, Huizhu Li^1^, Wenliang Kong^6^, Rui Chen^1,^*, Zhipeng Chen^1,^*

# 1.Supplementary experiment section

# 1.1 Synthesis of peptide

Peptide was synthesized by standard Fmoc strategy solid phase peptide synthesis (SPPS) on Rink Amide resin (ChemImpex, polystyrene matrix, 100-200 mesh, 0.47 mmol/g). Amino acid (3 equiv.) activation was performed with O-(benzotriazol-1-yl)-N, N, N’, N’-tetramethyluronium hexafluorophosphate (HBTU, 3 equiv.) and N, N-diisopropylethylamine (DIPEA, 4 equiv.) in dimethylformamide (DMF). Amino acids were coupled for 40 min at room temperature. Fmoc deprotection was performed using 4- methylpiperidine [20% (v/v) solution in DMF], while washing steps were performed with DMF and dichloromethane (DCM). For the synthesis of N-terminally derived DOTA-peptides, DOTA-tris(tertbutyl)-ester was used (2 equiv.), coupled to an activation with 2 equiv. of HBTU and 3 equiv. of DIPEA, with a coupling time of 1 h. Cleavage and side chain deprotection were performed using a mixture of trifluoroacetic acid (TFA)/triisopropylsilane (TIS)/water [95:2.5:2.5 (v/v/v)] at room temperature for 1.5 h. After vacuum evaporation, the crude peptide was precipitated in diethyl ether and lyophilized. After dissolution in an acetonitrile/water mixture [∼50:50 (v/v)], purification was performed by preparative reversed phase high-performance liquid.

# 1.2 In Vivo Skin Irritation (Draize Patch Test) and Histopathological Studies

Skin irritation study was conducted as described previously with some modifications^[34]^. The back of the mice was shaved (on) prior to the study. Then, the blank gel and nanocomposite hydrogel (100μL) was applied to the shaved area once daily for a period of 7 days. Mice treated with PBS served as a negative control. Twenty-four hours after the last topical administration, images of the mice backs were acquired and analyzed. Then, the mice were sacrificed, and the skin tissues were excised for histological examination. Afterwards, the tissues were fixed in 10% formalin, embedded in paraffin, and stained with H&E for further histopathological examination. Five mice were used for each test group.

# 1.3 Microcomputed Tomography Imaging

After the treatment, AIA mice of different therapeutic groups were imaged for whole body 360° scanning on an animal computed tomography scanner (Triumph X-SPECT/X-O CT, Gamma Medica-Ideas, USA), respectively. During the CT imaging, the mice were kept under anesthesia with isoflurane. CT imaging parameters were listed as the following: tube current = 270 μA, tube voltage = 80 kV, field of view = 80 mm × 80 mm, effective pixel size = 50 μm, and slice thickness = 154 μm. The scanned paws were reconstructed into a three-dimensional structure following the filtered back projection method. 3D bone volume including phalanges.

# 2.Supplematary Tables and Figures

**Table S1 Pharmacokinetic of TP in different groups.**

**Table S2 Histopathological scores**


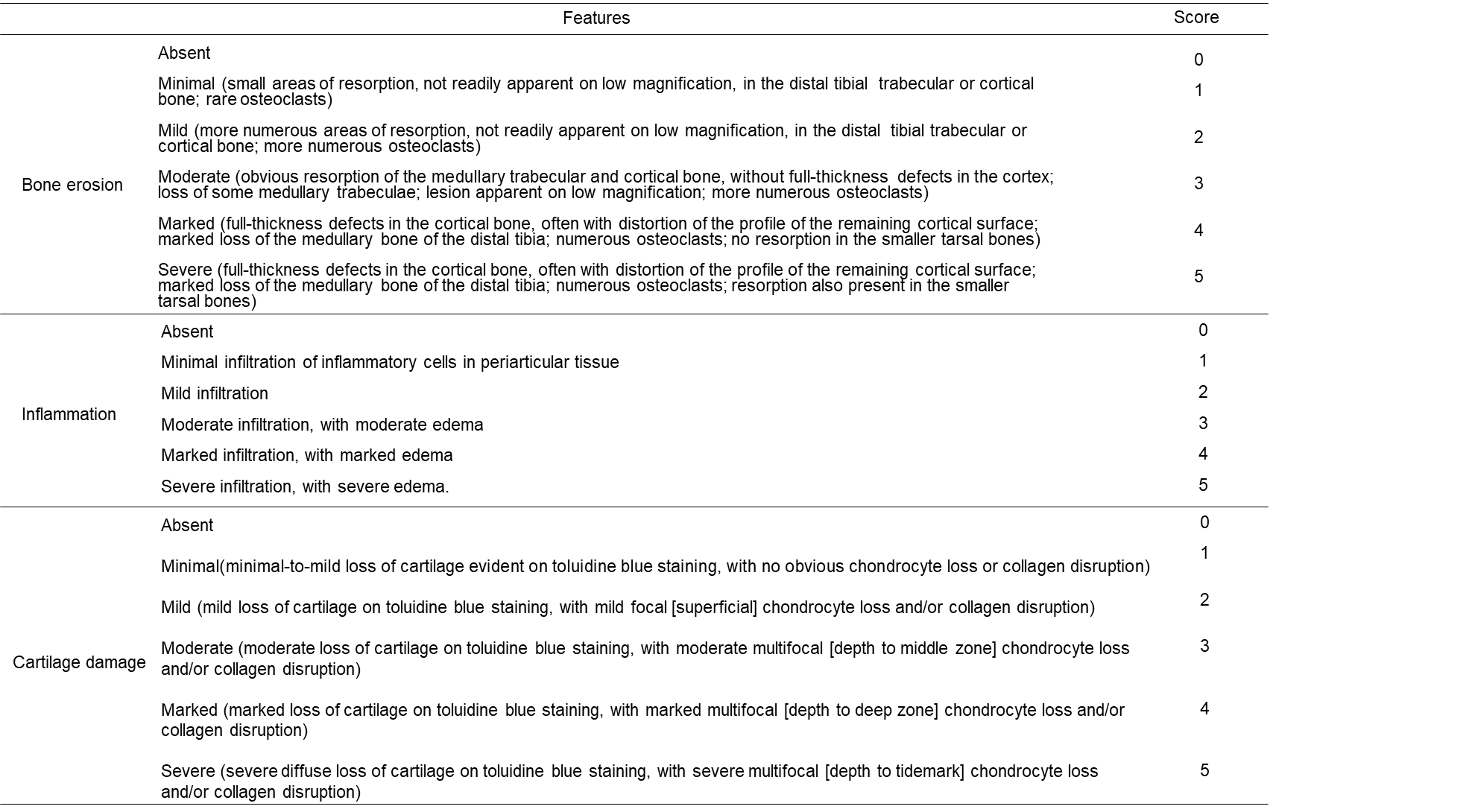


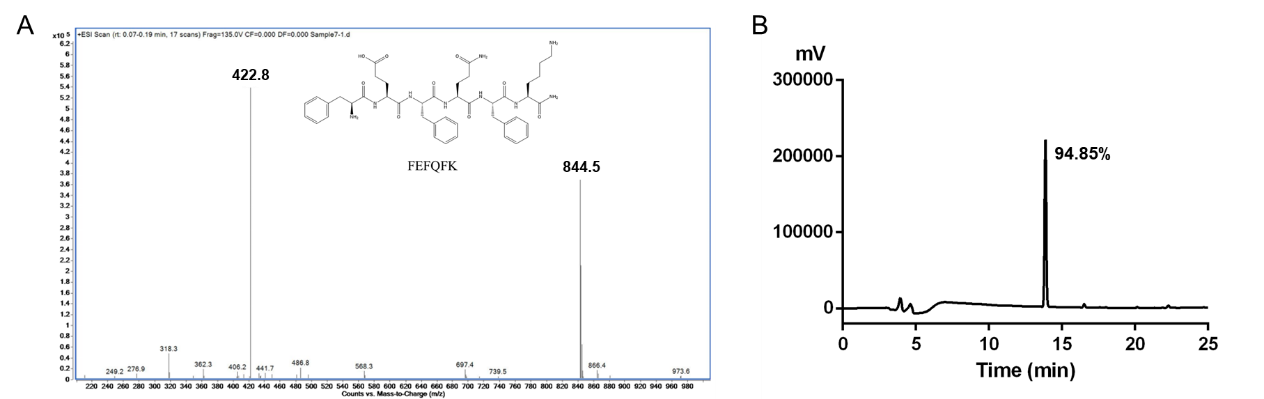


**Fig. S1 Molecular weight and purity verification of FEFQFK sequence.** (A) MALDI of FEFQFK, (B) Purity confirmation of FEFQFK by HPLC.

**
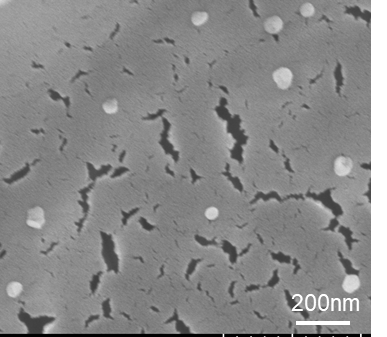
**

**Fig. S2 SEM of TP@HSA NPs.**


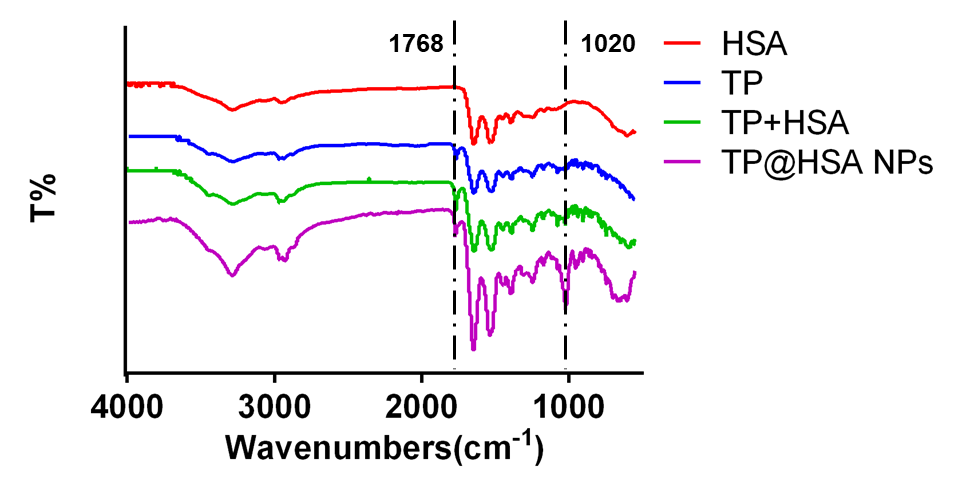
**Fig. S3 FTIR spectra of HSA, TP, the mixture of HSA and TP, and TP@HSA NPs.**


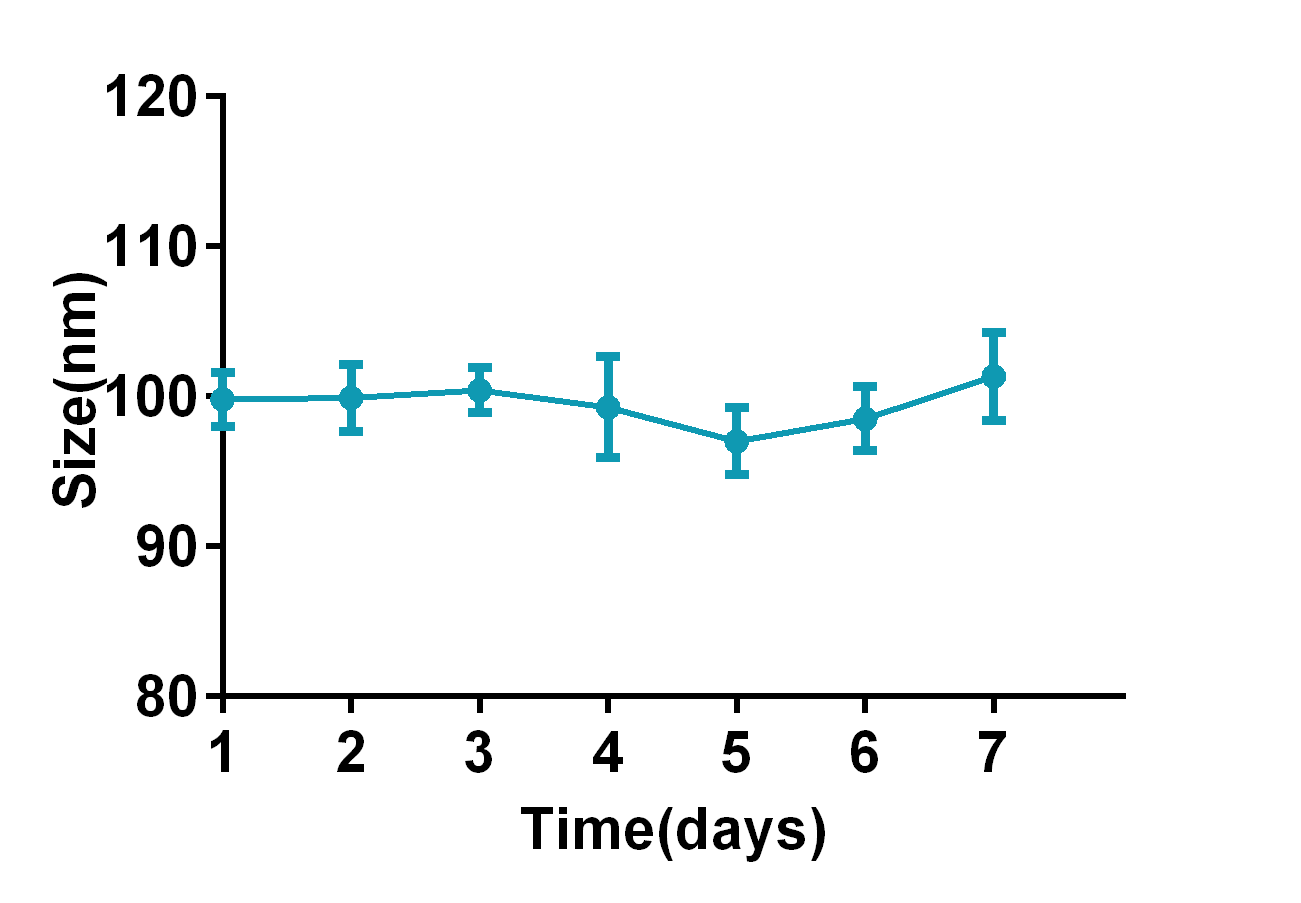


**Fig. S4 Study on the size stability of TP@HSA NPs.**


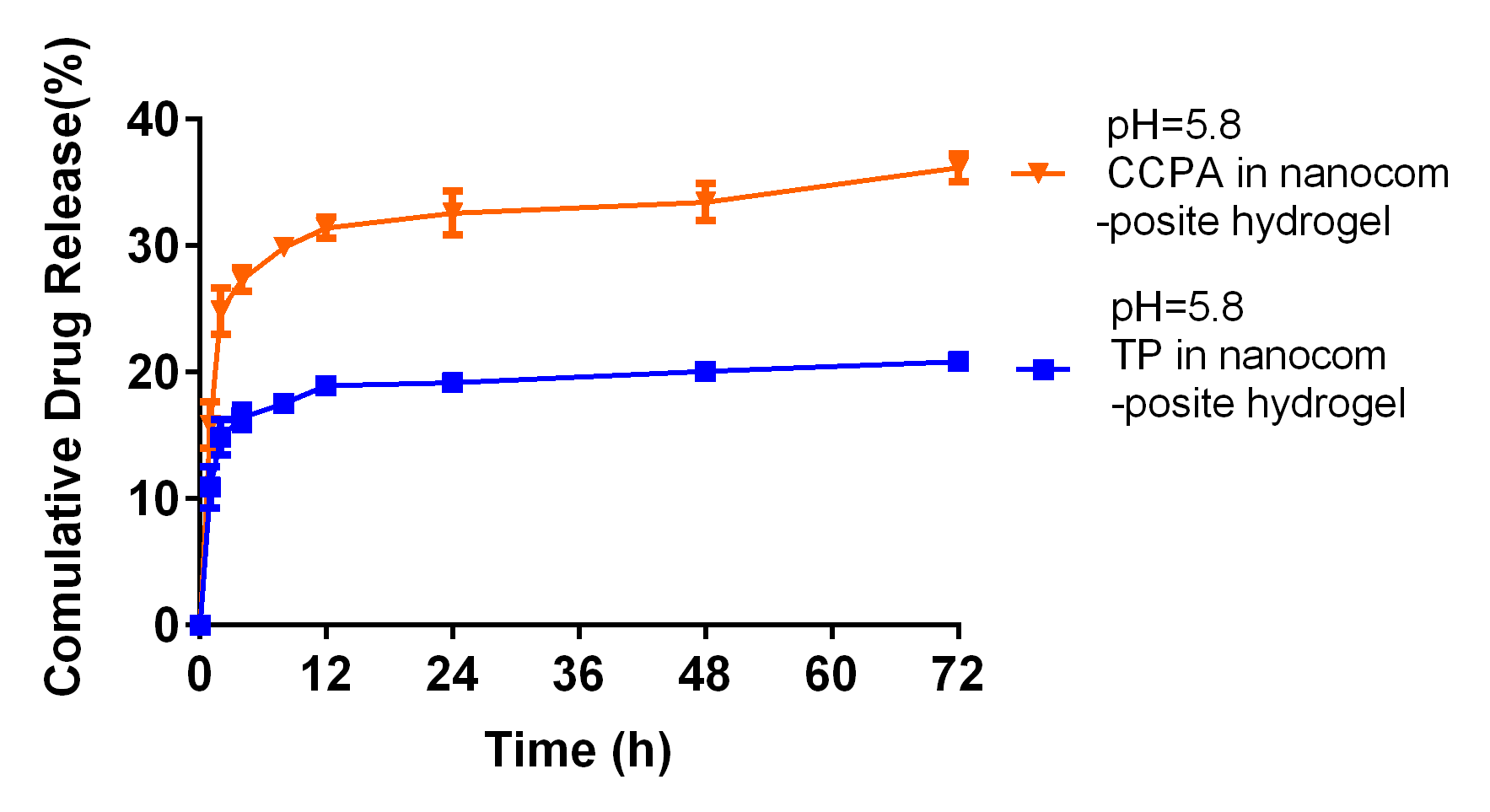


**Fig. S5 Comparative of the release behavior of CCPA and TP from nanocomposite hydrogel in different pH.**


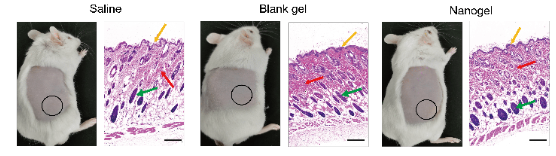
**Fig. S6 Histop****athological images obtained by H&E staining.** Yellow arrow represents stratum corneum, red arrow represents collagen fibers, green arrow represents hair follicle, scale bar=100 μm.

**
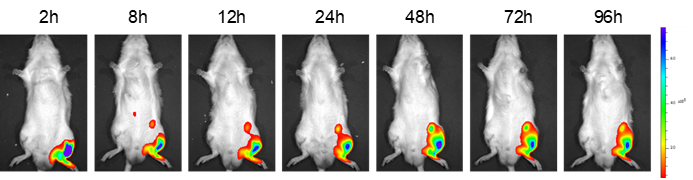
**

**Fig. S7 Imaging of A****IA mice treated with TP@HSA NPs in ST36 in left site at diﬀerent point-in-times.**


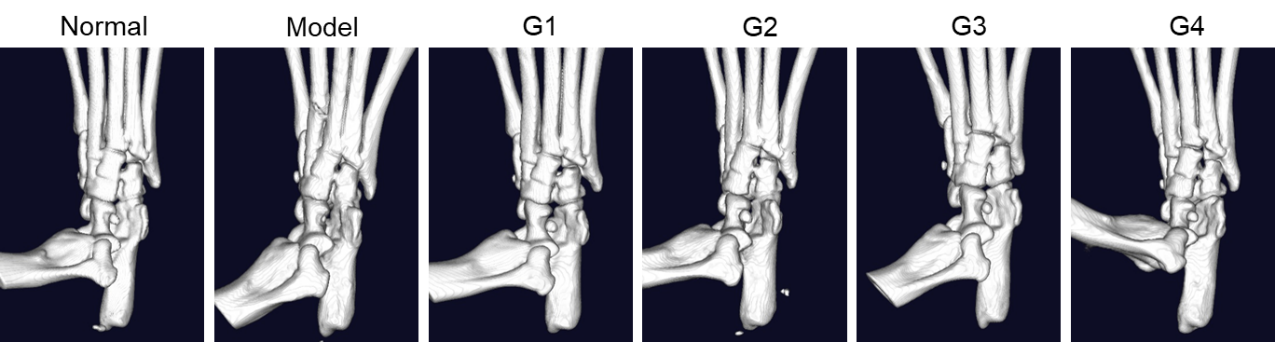


**Fig. S8 Representative micro-CT images of the ankle joints at day 28.**
